# Quantification provides a conceptual basis for convergent evolution

**DOI:** 10.1111/brv.12257

**Published:** 2016-03-01

**Authors:** Michael P. Speed, Kevin Arbuckle

**Affiliations:** ^1^ Department of Evolution, Ecology and Behaviour, Institute of Integrative Biology, Faculty of Health & Life Sciences University of Liverpool Liverpool L69 7ZB U.K.

**Keywords:** convergence, methods, homoplasy, parallelism, evolutionary ecology, phylogenetic comparative methods

## Abstract

While much of evolutionary biology attempts to explain the processes of diversification, there is an important place for the study of phenotypic similarity across life forms. When similar phenotypes evolve independently in different lineages this is referred to as convergent evolution. Although long recognised, evolutionary convergence is receiving a resurgence of interest. This is in part because new genomic data sets allow detailed and tractable analysis of the genetic underpinnings of convergent phenotypes, and in part because of renewed recognition that convergence may reflect limitations in the diversification of life. In this review we propose that although convergent evolution itself does not require a new evolutionary framework, none the less there is room to generate a more systematic approach which will enable evaluation of the importance of convergent phenotypes in limiting the diversity of life's forms. We therefore propose that quantification of the frequency and strength of convergence, rather than simply identifying cases of convergence, should be considered central to its systematic comprehension. We provide a non‐technical review of existing methods that could be used to measure evolutionary convergence, bringing together a wide range of methods. We then argue that quantification also requires clear specification of the level at which the phenotype is being considered, and argue that the most constrained examples of convergence show similarity both in function and in several layers of underlying form. Finally, we argue that the most important and impressive examples of convergence are those that pertain, in form and function, across a wide diversity of selective contexts as these persist in the likely presence of different selection pressures within the environment.

## I. INTRODUCTION

While much of evolutionary biology is interested in the creation and maintenance of diversity, there remains an important place for the study of phenotypic similarity, especially where this has evolved independently in different lineages. The evolution of phenotypic similarity is usually called convergent evolution and in early writings, convergent phenotypes were interpreted as similar outcomes of adaptation to similar environments (Muir, 1924; Mahler *et al.*, 2013). It is now recognised however that phenotypic convergence can have other explanations (Losos, 2011), such as genomic and developmental bias, similarity in phenotypic constraints and even mere chance (Stayton, 2008; Sanger *et al.*, 2011; Rosenblum, Parent & Brandt, 2014). Furthermore some species may evolve similar phenotypes but use them for quite different biological functions, in which case convergence has evolved in different functional contexts (Losos, 2011).

Although the concept of convergent evolution has been recognised and studied since Darwin (Darwin, 1859; Muir, 1924; Haas & Simpson, 1946) it is currently undergoing a resurgence of interest (see recent reviews in Arendt & Reznick, 2008; Leander, 2008; Conway Morris, 2008; Stayton, 2008, 2015; Lukes, Leander & Keeling, 2009; Christin, Weinreich & Besnard, 2010; Losos, 2011; McGhee, 2011; Scotland, 2011; Conte *et al.*, 2012; Maeso, Roy & Irimia, 2012; Martin & Orgogozo, 2013; Rosenblum *et al.*, 2014). Some of this recent interest is due to the continuing debate about the significance of convergence in limiting biodiversity. If evolutionary forces that result in convergence are prevalent, then the phenotypes of organisms may be relatively predictable, and biodiversity will be constrained (Conway Morris, 2003, 2008).

It has been argued that for many biological functions there are often limited engineering optima, in effect few ways to ‘do things well’. If such limitations are prevalent then adaptive evolution will repeatedly draw phenotypes towards similar forms (Conway Morris, 2008). There may for example be only a few types of morphology capable of enabling flight, and therefore the ‘phenotypic options’ available to produce flying organisms are limited. In addition there may be few cost‐effective ways to do some things well, so that even if there are a multiplicity of functional options for certain phenotypes, variable costs of implementation will limit the kinds of phenotypes which commonly evolve.

The diversity of life would also be constrained if there are frequent genetic homologies across species, biasing the range of genotypes on which mutation and natural selection can operate to shape the phenotype (Losos, 2011; Scotland, 2011). This type of constraint has usually been discussed in the context of what is known as ‘parallel’ evolution, in which phenotypes of organisms converge *via* independent mutations in similar genetical systems (Conte *et al.*, 2012). Hence, in parallel convergent evolution, natural selection operates on a relatively narrow subset of all potential genotypes (see Sanger *et al.*, 2011; Rosenblum *et al.*, 2014). The end result of any form of constraint, genetic or engineering, is a limitation in the phenotypic diversity of life.

A contrasting view, made famous by Stephen Gould (based on the same data from the Burgess Shale that Conway Morris used to come to the opposite conclusion) is that life is strongly influenced by stochastic events (so‐called ‘contingency’), which make phenotypes relatively unpredictable (Gould, 2000; see discussion in Powell & Mariscal, 2015). To repeat Gould's now familiar analogy, if we re‐ran the tape of life, very different forms of biodiversity would evolve each time. Answers to questions about the relative prevalence and causes of evolutionary convergence have profound implications for our understanding of the limits of biodiversity, and this has likely driven the increased attention given to the subject in recent years. Furthermore convergence is not just about natural evolution, it is now being recognised that artificial selection on agricultural organisms has led to repeated selection for the same kinds of traits causing convergent evolution at the phenotype and genotype levels (Lenser & Theißen, 2013). Hence the study of convergence may have important applications to our understanding of how humans cause organismal change during domestication.

The simplest way to identify phenotypic convergence is by reconstruction of ancestral states over a phylogeny so that independent, convergent, transitions to similar phenotypes can be recognised. Recent advances in molecular biology and phylogenetic computing have made the publication of suitable large‐scale phylogenies increasingly commonplace; hence the fundamental raw materials for the evaluation of evolutionary convergence are increasingly available. In turn this has led to the development of mathematical and computational tools to identify the presence and characteristics of convergence (Kluge & Farris, 1969; Muschick, Indermaur & Salzburger, 2012; Ingram & Mahler, 2013; Parker *et al.*, 2013; Arbuckle, Bennett & Speed, 2014; Stayton, 2015; Thomas & Hahn, 2015; Zou & Zhang, 2015). Furthermore new genome‐level datasets enable researchers to investigate the contribution of parallel evolution to convergence (Conte *et al.*, 2012) and to use methods from experimental evolution to test hypotheses about the predictability of phenotypes (MacLean & Bell, 2003; Fong, Joyce & Palsson, 2005).

At present the literature is strongly biased towards reporting demonstrations of convergence, in which similar traits are repeatedly identified across different lineages. A systematic example‐driven approach is given in McGhee's excellent recent text (McGhee, 2011), which brings together many examples of convergence at different levels of life and in different taxonomic groups. By contrast, the theoretical literature on convergence is much more limited (although see McGhee, 2001, 2011). This might be because no special ‘theory of convergence’ is necessary since convergence is widely understood to operate within the accepted frameworks of modern evolutionary biology.

In this review we argue that while we do not need special evolutionary theory to explain convergence, there is nonetheless room to refine the conceptual basis of convergent evolution. We attempt to make two points. First, convergent evolution must be quantifiable: if we have good tools to measure the frequency and strength of convergent phenotypes, we can perhaps begin to resolve what is in effect a profound debate about the frequency and strength of convergent evolution and hence the predictability of biodiversity. Although early cladistics techniques used various measures that indicated convergence, only relatively recently have statistical and computational methods been designed specifically to quantify convergence. We attempt to give a non‐technical overview of these methods here.

Second we consider what it means to say that some traits are ‘highly convergent and hence predictable’. To examine this statement we point out that it is necessary to consider the level of organisation of the phenomenon in question (at its very simplest, form *versus* function). Subsequently we argue that a strong test of the proposal that life is constrained and highly convergent must explicitly include evaluation of variation in environmental heterogeneity. Traits that are repeated across life in very many selective contexts (such as are likely present in different habitats) are almost certainly very highly constrained; traits that vary with local conditions are less so. By taking a quantitative approach to the different levels at which convergence can take place, we can begin to see a framework within which the predictability of life forms can be evaluated.

## II. MEASURING CONVERGENT EVOLUTION

We aim first to describe the kinds of measures that can be made of evolutionary convergence. We intend a non‐technical overview and hence we deliberately omit detailed accounts of methods. In the sections that follow, we focus first on quantitative methods and then briefly describe general approaches from genomics and experimental evolution. Before this, however, we discuss some general points which are broadly relevant to measuring convergent evolution.

### (1) Two general issues in the quantification of convergence

#### (a) *Considerations of scale and sample size in convergence measures*

Comparisons of any measure across different animal groups is made difficult by the fact that the scale is likely to differ between groups in multiple ways. By this we mean that in groups with a large number of species, the maximum possible number of convergent events is larger, and so we would expect more instances of convergence in larger groups just by chance. Similarly, in older groups there has been more time for phenotypic evolution to occur and therefore there is likely scope for more convergent evolution when measured quantitatively in older compared to younger lineages. Combined, this means that comparisons of measures of convergence amongst different trees require some form of standardisation. This could perhaps be achieved in some cases by calculating rates of a form such as ‘number of convergent events per species’ (although individual species cannot experience convergent evolution, such a standardisation still accounts for the fact that greater amounts of convergence may be expected in clades with more species) or ‘amount of convergence per million years or per species’ (such as used by Stayton, 2008), or calculating proportions of the measure compared to a theoretical maximum. A similar issue arises when considering convergence in multivariate phenotypes in that the more characters included in the analysis, the greater the potential number of instances and amount of convergence, leading to the same problems of standardisation across phylogenies and examples. The influence of sample size and the nature of the traits in question have recently been considered in detail in the context of homoplasy as relevant to phylogenetic reconstruction by maximum parsimony. This is an important technical issue and we direct the reader to two recent papers in particular for further information (Cuthill, Braddy & Donoghue, 2010; Cuthill, 2015).

#### (b) *Representation of cause in convergence measures*

Throughout this review, we often discuss convergence as an adaptive process and use examples of adaptive convergent evolution when summarising methods (simply because most, but not all, cases of convergent evolution are likely to have an adaptive basis). This in essence treats convergent evolution as the product of functional constraints, but Stayton (2008) has highlighted that the more characters included in an analysis, the greater the potential for finding convergence in some subset of those characters. Convergence may also be a result of phylogenetic rather than functional constraints (Wagner, 2000), such as we see in cases of ‘phylogenetic inertia/stasis’. Although the term phylogenetic inertia has been used imprecisely and defined variably in the literature (Blomberg & Garland, 2002), we use it here to refer to the pattern of phenotypic similarity within a lineage whereby clades are characterised by particular phenotypes regardless of the ecology of individual species. This implies either a slow enough rate of phenotypic evolution that adaptive change is difficult to detect or that the lineage is characterised by other traits than constrain the evolution of the trait under consideration in a given instance. As highlighted in later sections during discussions of particular methods, this ‘stasis’ in phenotypes is often difficult to disentangle from ‘true’ convergence, particularly where it is desirable to condense information about convergence into a single measure. In such cases, we stress that analysing the evolutionary history, such as estimating and visualising the ancestral states of the trait of interest is vital to understanding fully the evolution of convergence in any given system. Therefore analyses of convergence should typically be paired with broader investigations of the evolutionary history of the trait, which may often give some insight into whether the patterns we see are actually convergence or whether they better represent constraints due to phylogenetic inertia.

## III. MEASURES OF THE FREQUENCY OF CONVERGENT EVOLUTION

### (1) Convergence causes measurable inconsistencies in trees

Observing and recording evolutionary convergence is essential in evaluating the reliability of cladistic trees, hence the first measures of convergence emerged from early cladistic techniques. In fact cladistics often uses the more general term ‘homoplasy’ rather than convergence referring to any similarity between taxa in a phylogenetic tree which is not caused by descent from a recent ancestor. This includes convergent and parallel evolution but also reversal to an ancestral trait as a third category of homoplasy. All three may (or may not) be considered to be evolutionary convergence, depending on the precise definition of convergence used and the perspective taken (McGhee, 2011; Wake, Wake & Specht, 2011). High levels of homoplasy increase the probability of constructing a tree that does not reflect the true evolutionary relationships of the organisms within it, because phylogenetically distant species may be classed as descended from recent common ancestors based on phenotypic similarity that actually results from convergence (homoplasy) rather than shared derived characters (synapomorphies). David Wake, in his work on salamanders, wrote memorably of the difficulties caused by high levels of homoplasy: ‘The problem appears to be general; homoplasy is so common in salamanders that, despite many efforts, there is no generally accepted phylogenetic hypothesis for the order Caudata. Each hypothesis requires extensive convergence and reversal’ (Wake, 1991, p. 563).

One way to measure the presence of homoplasy is to evaluate the number of steps needed to construct a maximum parsimony tree. When a phylogenetic tree is constructed based on the values of a set of traits, convergence in the value of a trait between two or more members of the tree requires more steps to be added, because an evolutionary trait has arisen more than once. This makes the number of character changes in the tree (and hence its ‘length’) higher than it would be without convergence. Continuing this approach, researchers sought methods to find trees with the fewest number of evolutionary changes (the principle behind maximum parsimony methods) and so consequently the lowest number of convergent phenotypes. A number of early cladistic techniques were developed to quantify the excess number of steps caused by homoplasy and hence to measure homoplasy either as a trait on its own, or in an ensemble of traits (see Archie, 1996; Moore & Willmer, 1997). The best known is probably the ‘consistency index’ (Kluge & Farris, 1969). This measures the ratio between the number of steps in a fully parsimonious tree which has no convergence and the number of steps in a tree generated with maximum parsimony methods including assumptions about convergence for the trait(s) in question. As cases of convergence become more numerous, so the value of the ratio falls towards zero.

Subsequently various modifications and improvements to the consistency index have been suggested (see Table 1), including the ‘retention index’, which can loosely be thought of as a measure of the proportion of taxa that do not show convergence (see Farris, 1973, 1989; Archie, 1989) and the ‘homoplasy slope ratio’ which attempts to resolve index‐sample‐size biases (Meier *et al.*, 1991). Versions of the consistency and retention indices have been developed to evaluate quantitative traits and examples include the ‘quantitative convergence index’ of Ackerly & Donoghue (1998), and the more recent application by Klingenberg & Gidaszewski (2010) to evaluate the relationship between multivariate morphometric and phylogenetic data.

**Table 1:** A selection of phylogenetic methods to infer the frequency of convergent evolution. Unless stated each method is ‘process‐free’ in that no mechanism of convergence, such as adaptation, is assumed

Name of metric	Approach to measurement	Types of data	Limitations or other characteristics
Consistency index (CI) (Kluge & Farris, 1969)	Number of character state changes expected on tree/observed number of changes	Discontinuous traits, but see e.g. Klingenberg & Gidaszewski (2010) for recent developments for quantitative morphometrics	Requires parsimony approaches to tree construction
	CI decreases as homoplasy increases		Estimate of homoplasy increases with the number of taxa and characters (Archie, 1989)
			Sensitive to the number of autapomorphies (see Brooks, O'Grady & Wiley, 1986)
Retention index (RI) (Farris, 1973; see also Farris, 1989; Archie, 1989)	(Maximum steps on tree – observed state changes on tree)/(maximum steps on tree – state changes in data set)	Discontinuous traits, but see e.g. Klingenberg & Gidaszewski (2010) for recent developments for quantitative morphometrics	Requires parsimony approaches to tree construction
	RI decreases as homoplasy increases		Value can be inflated with number of taxa
Homoplasy slope ratio (Meier, Kores & Darwin, 1991)	Calculates a gradient indicating the number of extra steps to account for homoplasy, compares to value from a randomised data set	Binary characters	Requires parsimony approaches to tree construction
Phenetic *versus* phylogenetic trees (Couette, Escarguel & Montuire, 2005; Harmon *et al.*, 2005; Agrawal & Fishbein, 2006)	Homoplasy causes phenograms to deviate from independently derived phylogenies	Continuous data	Several statistical methods including Mantel test, and topology‐congruence statistics
	Assess deviations diagrammatically and statistically		
Pairwise distance–contrast plots (Muschick *et al.*, 2012)	Plot trait distance against phylogenetic distance, compared to predictions from Brownian motion	Continuous characters	Provides statistical identification and pictorial representation of convergence ‘hotspots’ and coldspots across axes of phenotype and phylogeny
SURFACE (Ingram & Mahler, 2013)	First uses Ornstein–Uhlenbeck processes to identify selective regimes	Continuous characters	Not ‘process free’
	Second uses AIC to reduce the number, providing a measure of number of convergence events		Assumes that convergence results from adaptive evolution
Phylomorphospace (Stayton, 2015)	Number of lineages that cross into a defined area of phylomorphospace and hence reside within a defined area of phenotypic similarity	Continuous characters	Sensitive to measure of morphospace that is identified as common in convergent species

The purpose of homoplasy indices is, however, to measure the reliability of trees derived through maximum parsimony methods, not specifically to measure homoplasy *per se* (Chang & Kim, 1996). Indeed tree construction may benefit from identifying and then downweighting traits with high levels of homoplasy (Goloboff *et al.*, 2008; Klingenberg & Gidaszewski, 2010). Such methods are, understandably then, likely systematically to bias the estimation of the amount of convergence since in practice they make use of trees built with the aim of minimising convergence. Furthermore, these measures were designed for use with maximum parsimony trees, but most modern trees (especially on large data sets) are constructed by maximum likelihood or Bayesian approaches. However, it is worth pointing out that methods based around ideas of ‘consistency’ have the advantage that they can often be developed for any data type from which a phylogeny can be built and so, unlike many other methods, they are not necessarily limited to evaluating convergence in just categorical or just continuous traits.

### (2) Methods designed to evaluate convergence

More recently, methods have been proposed to quantify convergence as a phenomenon in its own right (see Tables 1 and 2 for summaries of these techniques). These methods aim to elucidate different aspects of convergence rather than simply attempting to correct for it during the reconstruction of phylogenies. In essence, they move the field forwards by providing ways to tackle questions directly which were unable to be addressed with older methods. Most of these methods only work with quantitative, continuously variable traits. We describe these first and then briefly consider the issue of convergence in binary and categorical traits.

**Table 2:** A selection of phylogenetic methods to infer the strength of convergent evolution

Name of metric	Approach to measurement	Types of data	Limitations or other characteristics
Patristic/phenetic ratios (Stayton, 2008)	Calculate all pairwise ratios of (patristic distance/phenetic distance) in a tree	Continuous	‘Process‐free’ in that no assumed mechanism of convergence is required
	High values indicate convergence		
Wheatsheaf index (Arbuckle *et al.*, 2014)	Using distance matrix, find average distance between traits for members of a focal group, and of the set overall	Continuous, or sets of categorical traits assessed for frequency	Generally applied with adaptive convergence in mind (although this is not a necessity)
	Represent phylogeny by increasing trait difference values to the extent that they lack phylogenetic independence		Uses bootstrapping approach to evaluate how structure of a tree affects likelihood of identifying convergence
	Higher index values represent higher convergence levels		
Distance measures (Stayton, 2015)	Comparisons across two lineages	Continuous	‘Process‐free’ in that no assumed mechanism of convergence is required
	Compare phenotype distance of putative convergent species with that of the most divergent species between the lineages		

#### (a) *Phenetic versus phylogenetic trees*

**Figure 1:**
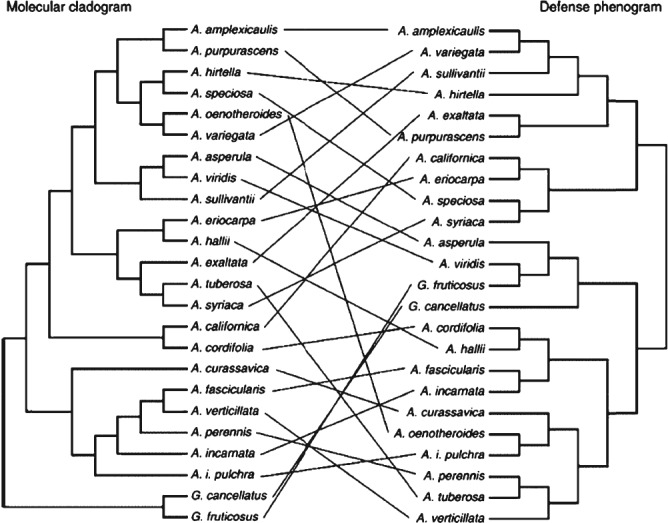
Tanglegram comparing a molecular phylogenetic tree (left) with a phenetic tree of defensive traits (right) for a set of plant species. Lines between trees link the same species and crossing lines indicate a lack of similarity in the two trees (e.g. where phenotype is more similar than implied by phylogeny, indicative of convergence). From Agrawal & Fishbein (2006), reproduced with permission of the authors and publisher.

An intuitive approach is to compare a phylogenetic tree and a corresponding phenetic tree (or phenogram) constructed using multivariate (quantitative) phenotypic data not used in the construction of the phylogenetic tree. If convergent evolution is common then the phenogram will tend to cluster species that are not grouped into clades in the phylogenetic tree. This ‘phenetic *versus* phylogenetic’ approach has been applied in diverse contexts including the evaluation of correlations in anti‐herbivore defences in plants (so‐called ‘defense syndromes’, Agrawal & Fishbein, 2006) and in evaluation of morphometric data from the skulls of New World monkeys, see Couette *et al.* (2005) who provide detailed advice on construction of appropriate phenograms. Visual comparison between phenetic and phylogenetic trees is an obvious first step, for example in Fig. 1 (taken from Agrawal & Fishbein, 2006) which shows that the structure of the molecular cladogram is not well reflected in the defence phenogram, which clusters species together on the basis of (convergent) phenotypic similarities.

It is possible to quantify the difference in topology between phenogram and phylogenetic tree, for example using topology‐congruence statistics (Shimodaira & Hasegawa, 1999; Agrawal & Fishbein, 2006). Alternatively, Couette *et al.* (2005) generated distance matrices for both kinds of tree and applied Mantel's Z test (Mantel, 1967) to evaluate the presence of an association between the structure of the two matrices. This approach was also taken by Harmon *et al.* (2005) in their multivariate examination of convergence in *Anolis* lizard morphometrics.

These statistics evaluate the (dis)similarity of species relationships in phenotypic and phylogenetic measures; they do not necessarily indicate convergent evolution as the cause. For example, phenotypes that are neutral with respect to selection and have been influenced significantly by drift, or that typically faced divergent selection pressures, can also produce phenograms that deviate markedly from the corresponding phylogenetic tree without showing convergence. An issue with this approach is that where there are few instances of convergence they may not be recognised as such. We note that simulation studies may help to assess how powerful such a method would be for identification of convergent evolution.

However where tree topology is not the same (or distance matrices are not highly associated), and convergence has been identified, then this approach could (we believe) be adapted to ask whether individual traits contribute more or less to the signal of convergence. For organisms in a given phylogeny, alternative phenograms can be constructed for alternative sets of morphometrics, for example systematically removing one trait at a time and measuring topology congruence or using Mantel's test. As convergent evolution becomes more common, for example, so the correlation in the Mantel's test should decrease. In their discussion of exudate‐feeding in New World monkeys, Couette *et al.* (2005) describe several associated adaptations, including changes in gut structure, teeth, and claws. Perhaps some of these traits are more frequently convergent than others, in which case including them causes Z scores to be lower, and calculating bootstrapped 95% confidence intervals for the Z scores could provide a means of adequately comparing the scores for each trait. This approach can be used with any type of data from which distance trees can be constructed, including multivariate data, but care must be taken to ensure that different topologies reflect convergence to some extent, perhaps by visual inspection.

In summary this is a relatively simple approach which may help researchers understand the relationships between phylogeny and phenotypes, and in some cases enable quantification of the role of each phenotype in the convergence of a set of functionally related phenotypes.

#### (b) *Pairwise distance measures*

**Figure 2:**
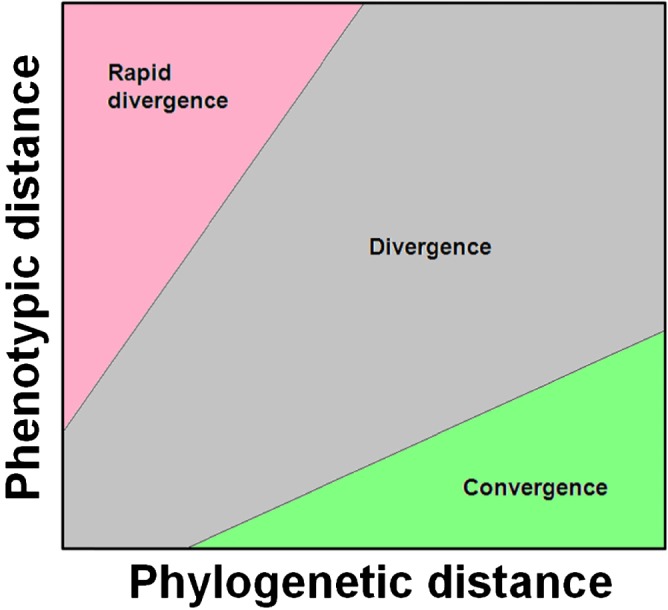
Representation of the plot‐space used by the pairwise distance‐contrast method. This method plots phylogenetic distances against phenotypic distance and the results are broadly interpreted as in the differently shaded regions. Convergence (or stasis) is considered when there has been little phenotypic divergence over large phylogenetic distances (the area in green).

Muschick *et al.* (2012) tested whether (quantitatively measured) morphological convergence was exceptional in African cichlid fish. They devised a novel method which they term ‘pairwise distance‐contrast plots’ and which effectively makes use of the prediction that, under convergent evolution (or, incidentally, evolutionary stasis), we expect to find relatively little morphological difference compared to the phylogenetic distance between a given pair of species. Muschick *et al.* (2012) therefore simulated phenotypic evolution under a null model (such as Brownian motion) and compared the difference between the position of these simulated data and the observed data on a plot of phenotypic *versus* phylogenetic distance (Fig. 2). Convergence is inferred when observed data fall more often in a region of lower phenotypic but higher phylogenetic distance than expected based on the simulations (the green area in the lower right of Fig. 2). This method has the advantage of allowing the direct comparison of the observed and the predicted distributions from evolutionary null models, but cannot differentiate convergence from stasis. It makes good use of a basic prediction of convergent evolution to provide a visual and statistical assessment of similarity between species in a single trait or set of traits. Finally, the distance–contrast plot method can be easily co‐opted to test for the presence of particularly fast divergence, as (for instance) might be expected from adaptive radiations with strong disruptive selection between recently diverged species.

#### (c) *Selective regimes*

An alternative approach for the identification of adaptive convergence of quantitative traits using ‘selective regimes’ was proposed by Ingram & Mahler (2013). They called their method SURFACE [a recursive abbreviation of ‘SURFACE Uses Regime Fitting with Akaike Information Criterion (AIC) to model Convergent Evolution’] and it is implemented in an R package of the same name. This is based on the methods described by Hansen (1997) which model the effects of selection and drift across phylogenetic trees using Ornstein–Uhlenbeck (OU) processes, which are arguably a better approximation to evolution of phenotypes under convergence than the Brownian motion processes that are assumed by many phylogenetic methods.

**Figure 3:**
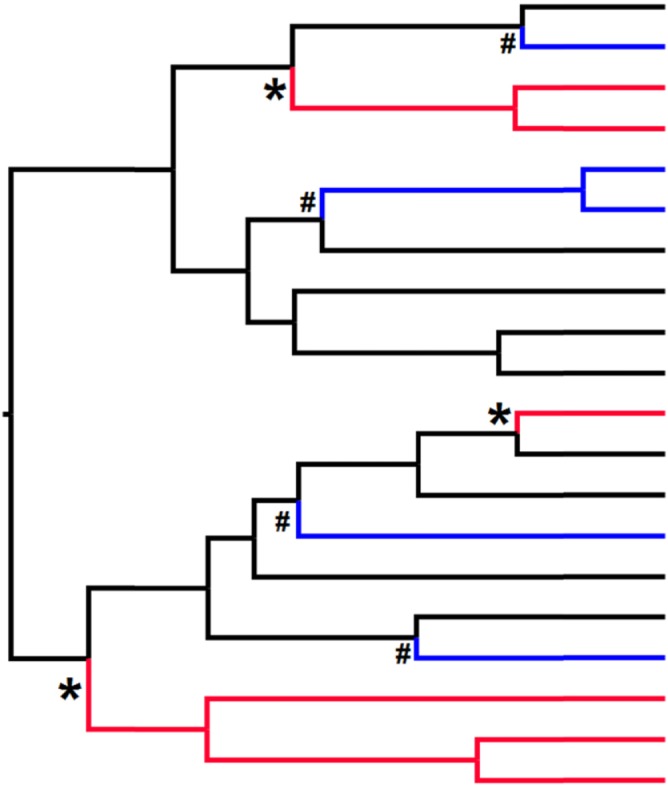
Graphical representation of output from SURFACE analyses. In this case there have been convergent shifts to both the red and blue regimes (black branches represent the ancestral regime). More specifically, the blue regime has arisen on four separate occasions (marked by #), and the red regime has arisen on only three separate occasions (marked by *), despite containing more contemporary species than the blue regime.

OU models represent the constrained evolution of a trait which evolves around a particular ‘optimum’ value, called a selective regime. If the optimum shifts (such as by adaptation to a different selection pressure) the trait then evolves around this new value, and a new regime is represented in the phylogeny. Figure 3 illustrates alternative regimes ‘painted’ across a hypothetical tree in the same manner as described in Ingram & Mahler (2013). In this figure, * and # represents the presence of two convergent regimes (selection for different trait values) that evolve independently more than once in the tree and are denoted as red and blue branches, respectively (black branches represent the ancestral regime).

SURFACE first performs a ‘forward’ phase in which it finds and ‘paints’ on alternative selective regimes to different branches as illustrated in Fig. 3. For a phylogeny with *n* branches, SURFACE first fits models with 1 to *n* different selective regimes selecting the best estimate of the number of alternative selective regimes using AIC. This is followed by a ‘backward’ phase which compares the resulting selective regimes to each other, essentially asking which of them are sufficiently similar to be classed as repeated evolution of the same regime (which is considered evidence for convergent evolution). Again AIC methods are used to evaluate the best of the alternative backward phases and simulated data can be used to test statistically whether the number of observed convergent regimes is greater than expected by chance. Hence SURFACE can evaluate whether traits are convergent and how often convergence is estimated to occur within a given phylogenetic tree. This method requires quantitative data, and Ingram & Mahler (2013) suggest that at least two traits are used in any analysis.

#### (d) *Simple distance and phylomorphospace approaches*

Stayton (2008, 2015) proposed at least two methods for ascertaining convergence frequency. He originally suggested a method in which pairs of taxa could be classified as convergent if they were more similar than their ancestors (Stayton, 2008), and proposed weighting methods to account for phylogenetic distance between species. More recently Stayton (2015) outlined an approach in which species are represented graphically in a plot of morphological space, and a limited area of this is defined as the focus of potential convergence. All the members within this ‘focal morphospace’ are phenotypically similar. The phylogenetic connections between species are then represented in what is termed a ‘phylomorphospace’. The number of convergent species is counted as the number that reside within the focal morphospace *and* belong to lineages that cross the boundary of the morphospace to enter it. This indicates convergence from outside of this phenotypic space, whereas members of clades contained within the morphospace would not be counted as convergent. This is conceptually simple, which is appealing, and does not rely on assumptions about causes of convergence such as adaptation (as is assumed, at least implicitly, by some other methods such as SURFACE). However the phylomorphospace approach does have the limitation that the values obtained by this approach will vary with the method used to define the focal morphospace itself (see discussions in Stayton, 2015).

## IV. MEASURING THE STRENGTH OF CONVERGENCE

### (1) Phenotypic similarity *versus* phylogenetic distance

Measuring the presence or absence of convergent evolution removes subjectivity and can provide evidence of the frequency of convergence in different traits, or in different environments. After a method such as SURFACE indicates convergence, this raises the possibility of posing a different question – are the characteristics of some types of convergent traits more similar than others? In effect, is evolutionary convergence stronger with some kinds of phenotype than with others?

Stayton (2006) proposed methods involving the use of similarity of species within a phenotypic space and also movement within this space between ‘ancestral’ and ‘descendant’ species. Although initially presented as a way of identifying convergent evolution based on either of three evolutionary patterns we might expect from convergence (using permutation tests to determine significance), the methods could feasibly also be used to provide a quantifiable measure of convergence. For instance Stayton (2006) provides information on the variance in phenotype within particular groups (herbivorous lizards), and this could potentially act as a metric of convergence over and above its use as a test statistic to be assessed by permutations.

Stayton (2008) later proposed a conceptually simple metric to determine what we term the strength of convergence. He proposed that for all permutations of taxon pairs in a tree the ratio of (patristic distance/phenetic distance) is calculated. High values would tend to indicate strong convergence, since this implies either small phenetic distances and/or large patristic distances. These ratios could be used in different ways, for example, to identify putative convergent pairings by their large values, or averaged across trees to see if some kinds of trait were on average more convergent than others.

### (2) Use of ‘focal’ groups to test hypotheses of convergence strength

To examine the issue of strength of convergence we (Arbuckle *et al.*, 2014) proposed a related conceptual framework for measuring more directly the ‘strength’ of convergent evolution and a method to test this quantitative aspect of convergence (the ‘Wheatsheaf index’). In this method we first define a subgroup (the ‘focal group’) of species which exist within a similar niche, or exploit their environments in similar ways. Suppose, for example, we consider adaptive respiratory specialisations in diving animals. We can find a number of putatively adaptive traits associated with uptake and storage of respiratory oxygen: relative muscle mass, lung volume, myoglobin concentration, solubility of myoglobin, amino acid charge in myoglobin and the number of amino acid substitutions of a particular type in the molecule (Mirceta *et al.*, 2013). We may be able to investigate which – if any – of these traits are most strongly convergent in diving mammals. In other words, which traits have the most similar values after accounting for phylogenetic relatedness of species across the relevant tree.

**Figure 4:**
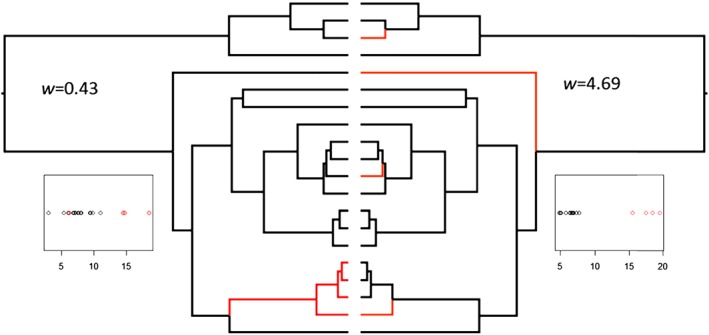
Diagrammatic example showing situations that would result in relatively low (left) and high (right) Wheatsheaf (*w*) index values for a given tree. In this example there are data for a single trait (inset plots show distribution) for 20 species overall, 16 non‐focals (black tips) and four focals (red tips). A lower index results from closely related focal species with trait values that overlap with non‐focals. By contrast, a higher index results from more distantly related focals with highly distinct trait values from non‐focals.

The value of the Wheatsheaf index increases as members of the focal group become more similar to each other, and as the focal group becomes collectively more divergent from the set as a whole (implying a greater shift from one region of phenotypic space to the other). However, phenotypic similarity within the focal group is penalised by phylogenetic distance, so that more weight is given to phenotypic similarity in distantly related species than those recently diverged from a common ancestor (as the latter are expected to be more similar by chance due to shared ancestry rather than convergence). Figure 4 provides a graphical representation of how changing the distribution of a trait in the focal group and its phylogenetic distribution alter the index. Branches in red are members of the focal group, the inset plots show the trait values (separated into red focal and black non‐focal species), and the Wheatsheaf index is given as *w*. As can be seen, higher index values are observed as the focal species are more distantly related to each other and as their trait values become more similar to each other and more distinct from non‐focal species.

The Wheatsheaf index can be used quite flexibly, and can be generated for a single quantitative trait or for sets of traits summarised by Euclidean distances from each other. Note that a wide range of trait types can be used, including continuous, count data, or a suite of binary traits (although not single binary traits). In effect, the Wheatsheaf index can be employed for any (set of) trait(s) for which a meaningful phenotypic distance can be generated (and can easily be modified to use distances other than Euclidean such as Manhattan or Procrustes distances if desired). A bootstrapping approach can account for phylogenetic constraints imposed by the topology of a given tree (which can, for instance, limit the amount of convergence possible) and enable generation of a *P*‐value evaluating the null hypothesis that phenotype convergence is no stronger than expected by chance. In the well‐known convergent ecomorphs in *Anolis* lizards, application of the Wheatsheaf index indicated that the grass–bush ecomorph shows exceptionally strong morphological convergence even in comparison to other ecomorphs (Arbuckle *et al.*, 2014).

Serial application of the index across a set of traits may indicate which (if any) are more phenotypically convergent than others. In our diving mammals example, we could for instance speculate that increasing oxygen binding capacity of myoglobin by increasing its molecular charge is an easy and inexpensive way to increase muscle oxygen uptake. It might therefore be a more ‘universal’ and more strongly convergent respiratory trait in diving mammals than other more expensive or less effective phenotypes (Mirceta *et al.*, 2013). Hence, we would gain some texture to our comprehension of convergence: rather than simply asking whether convergence is important, we would be able to assert whether it is more or less important for certain kinds of trait. This in turn could provide data to begin to resolve the debate over the predictability of evolution, since the answer may be both yes and no, depending on the traits (or types of traits) being considered.

However, because as originally envisaged the Wheatsheaf measure of convergence strength compares values between a focal convergent group and the remaining non‐focal organisms, without modification it cannot be used for traits that are only present in the convergent organisms. For example, we may want to know how strongly convergent are forms of the camera eye, which is found in various vertebrates and invertebrates including jellyfish, annelid worms, molluscs and arthropods (Land & Nilsson, 2012), but the phenotype values for any organism outside of the ‘eyed’ group is zero and the focal/non‐focal ratio on which the Wheatsheaf index is based is therefore meaningless.

A potential workaround is to modify the question being asked and now to consider convergence within the set of organisms that contains the camera eye. We can examine the presence/absence of each component of the camera eye across all organisms in this set, to determine whether all structural components are convergent. In addition we could apply the Wheatsheaf index using all camera‐eyed organisms as the total set examined, but then taking subsets as the focal group those which exist in similar environments (aquatic or terrestrial; low or high light intensities), or use their eyes for similar purposes, such as predation or protection from predation (Land & Nilsson, 2012). In essence, this is a question of ensuring that the total group of organisms is well chosen to answer the questions: specifically, what should we consider as the non‐focal group?

Note that Stayton (2015) has recently pointed out that the Wheatsheaf Index may give similar values for examples of evolutionary stasis as for convergence. In stasis examples, the focal group may retain an ancestral state, and the remaining organisms diversify; now the focal group members are more similar to each other than to the group as a whole, but not because they have evolved together. We agree with this and it helps us make a point about this and other convergence measures: it is always desirable to investigate the ancestral states, and indeed the pattern of trait values across phylogenetic trees. Focusing only on the value of an index can be misleading.

### (3) Distance‐based measures of the strength of convergence

Stayton (2015) has recently proposed some simple metrics for measuring convergence strength based on the amount that two species converge phenotypically compared to selected species in their lineages. The metric is derived from the idea that ‘convergence occurs when two taxa evolve to be more similar to one another than their ancestors were to each other’ (Stayton, 2008, 2015, following Haas & Simpson, 1946). In one of his metrics he proposes an elegantly simple method that compares the difference in phenotype values of two putatively convergent species (*D*_tip_) with the maximum phenotype differences between another pair of species in their two lineages (*D*_max_). In his simplest metric, Stayton (2015) suggests calculating an index of convergence strength (*C*_1_) as follows,

(1) $$C_{1}=1–\left(D_{\text{tip}}/D_{\max}\right)\text{.}$$

Convergent species that are phenotypically similar, but come from lineages with large phenotype differences will generate a higher index value than those that (*i*) are phenotypically more dissimilar (larger *D*_tip_) or (*ii*) come from lineages with smaller phenotype ranges (*D*_max_).

Interestingly, Stayton (2015) suggests that any species extant or ancestral, can be represented in the denominator species pairing, since this value represents ‘the maximum distance that has been closed’. Some researchers may wish to limit this pairing to actual ancestors (rather than say extant sister species), thereby ensuring that the value measures the amount of evolved phenotype change between two lineages which is convergent. However as Stayton (2015) points out, if ancestral state reconstructions are used to estimate ancestral phenotypes, then *D*_max_ will take values lower than those recorded from extant species, and the convergence estimate (*C*_1_) will be conservative.

Where more than two species are being considered within a clade, Stayton (2015) suggests that either a representative species is taken from each clade or the inferred phenotype of the common ancestor may be taken. In the case of more than two lineages, he suggests that the average *C*_1_ value of all comparisons could be used to measure convergence.

The value of this simple index is a ratio constrained by the value of *D*_max_, so that a small absolute convergent change (*D*_tip_) could be a small or a large proportional change depending on *D*_max_. Stayton (2015) therefore proposed additional metrics which express convergence as a proportion of all phenotypic change in a lineage or a clade.

We note the interesting differences between these two approaches to convergence strength. The Wheatsheaf index (see Section IV.2) identifies an ecologically distinctive focal subset of species from the whole set under consideration, and asks if they are more similar to each other than the set as a whole. By contrast, Stayton's (2015) distance methods have no requirement for *a priori* ecological distinctiveness. We could choose any species pair here and ask whether they are more similar than the most extreme species contrasts between lineages. Furthermore, this distance‐based measure is not reliant on any specific process to explain convergence, it merely focuses on phenotypic similarity *per se*. The Wheatsheaf index, by contrast, will generally be used to test hypotheses about the strength of adaptive convergence (although not always, such as when previously identified convergent taxa are used as the focal group).

## V. THE ISSUE OF BINARY TRAITS IN CONVERGENCE MEASURES

One conceptual and methodological challenge is how (or whether) we can measure convergent evolution in single binary traits (e.g. presence/absence, yes/no, or by extension nominal categorical traits of more than two classes). We could use methods such as ancestral state reconstruction to consider the distribution of a convergent trait with respect to another trait which the first is purportedly convergent for. We could hypothesise for instance that use of camouflage in prey species is convergent in diurnal species due to limitations of predator visual systems. In this case we would look for the frequent evolution of camouflage within diurnal lineages. This could be formalised to some extent using methods developed to test for the correlated evolution of binary traits, such as Maddison's concentrated changes test (Maddison, 1990) or Pagel's test based on transition rates (Pagel, 1994), although correlated evolution is not exactly the same as convergent evolution and recent criticisms of tests of phylogenetic correlations would apply here also (Maddison & FitzJohn, 2015).

Another potential approach (related to these two tests) would be to compare values at the tip of the phylogeny (e.g. species) rather than the lineages through time. In this case, we would consider our observations of diurnal *versus* nocturnal species as fixed. Then, we could estimate transition rates of our convergent character (camouflage) over the tree and use these rates to simulate a binary trait many times, resulting in a ‘null set’ of distributions of crypsis at the tips. We could then compare how often the observed level of convergence at the tips is found in the simulated data sets, either generally in the number of independent origins of crypsis or specifically in the number/proportion of them that end up in diurnal species.

It should be noted, however, that such measures are unable to quantify the strength of convergence in any meaningful way for binary traits. By their very nature, single binary traits are either present or absent, and therefore they are either present in two lineages (i.e. convergent) or not.

## VI. INSIGHTS FROM SELECTED MOLECULAR STUDIES

This area has been well reviewed recently (Arendt & Reznick, 2008; Maeso *et al.*, 2012; Rosenblum *et al.*, 2014). Hence, we describe a brief selection of methods here and direct interested readers to these reviews.

The very‐large‐scale availability of genomic data is relatively recent but none the less a large number of studies have already examined genomic data sets for signatures of convergent evolution. What limits many studies (in our view) is the absence of equivalently large‐scale data sets on relevant phenotypes. In this light a promising approach to evaluate the frequency of parallel evolution has been proposed using data from studies in which phenotype differences between alternative populations are accounted for using, for example, whole‐genome quantitative trait locus (QTL) methods or candidate gene approaches. Conte *et al.* (2012) used a selection of case studies to evaluate the likelihood that orthologous gene(s) were independently used in creating the same phenotype in different populations. Where orthologues are not involved but phenotypes had evolved similarly anyway they could infer more general convergent evolution at different loci. In their study the estimated frequency of parallel evolution was 0.32 in QTL studies, and 0.55 for candidate gene studies. Ingenious as it is, this approach may at present be ahead of its time in the sense that usable data sets are not yet numerous: from an initial list of over 1600 papers, only 25 studies met the criteria for inclusion that Conte *et al.* (2012) set. This limits the capacity to look for convergence between both phylogenetically close and phylogenetically very distant species. When the number of published studies increases to a sufficient level, we may begin to be able to quantify the significance of parallel evolution on a broad evolutionary perspective using this kind of approach.

Where detailed phenotypic data are perhaps absent, but large‐scale genomic data are present, researchers may look for signatures of convergence by examining genomes of organisms within a functionally convergent focal group. Merhej *et al.* (2009) compared genomes of obligate intracellular bacteria, finding the repeated loss of 100 orthologous genes, whose functions are replicated within the host genomes (see also the review in Rosenblum *et al.*, 2014). However at the time of writing, some methods for quantifying molecular signatures of convergence from sequence data are proving controversial. For example, Parker *et al.* (2013) examined amino acid substitutions in more than 2000 orthologous genes within a sample of echolocating mammals (cetaceans and bats). By their methods, they found signatures of sequence convergence at more than 200 loci, and these unexpectedly included many genes apparently unrelated to echolocation such as genes involved in vision. However researchers have subsequently questioned the methodology which led to this conclusion, arguing that in fact there is no signature of convergence in this case (Thomas & Hahn, 2015; Zou & Zhang, 2015). In short, these criticisms largely revolve around the fact that the measures used by Parker *et al.* (2013) are not actually measures of convergence, and that the null model they employed for their significance tests was not appropriate. Thomas & Hahn (2015) and Zou & Zhang (2015) reassessed the data using more appropriate methods and tests and both found that there was no more genomic convergence than expected by chance, contrary to the conclusions of the original study.

Another promising approach is to employ methods of experimental evolution in microbes, which have short generation times and sufficiently large population sizes that mutants will be relatively plentiful and hence evolution is rapid (see recent review in Achaz *et al.*, 2014). In many experimental evolution studies, parallel populations are set up from a common ancestor and allowed to evolve independently with respect to a novel environmental challenge. Subsequently, phenotypes are measured, convergence is identified, and there is often then an attempt to identify the genomic changes responsible. Some researchers present alternative nutritional regimes to microbial populations, using ‘Biolog’ plates in which each well can contain a unique carbon source to which the microbial population must adapt. Multiple parallel populations can evolve alongside each other in sets of 96 well plates (MacLean & Bell, 2003). In one of a growing number of examples, Fong *et al.* (2005) found microbial populations evolving similar growth phenotypes on lactate or glycerol minimal media but, as evidenced by mRNA transcriptomics, often by diverse genomic routes. This provides evidence of convergence in function that is not paralleled at the genetic level.

Recent developments in molecular and genomic techniques therefore produce a variety of methods for examining convergent evolution. At the time of writing, some very promising approaches are at an early stage of development. However, we would make the point that we could apply some of the statistical methods described above at the molecular level so long as quantitative or semi‐quantitative traits can be measured. If, for instance, we can reconstruct the evolutionary relationships between protein molecules which are derived from a common ancestor, then we could look for signatures of convergence based on a focal subset (e.g. those functioning within cells *versus* those functioning between cells). In other words, rather than considering convergence amongst species at higher levels, we could compare convergence in the molecular structure of proteins that perform similar functions. Traits can include, for example, substrate binding affinity in enzymes. Such applications are fundamentally similar to that in the preceding sections if we consider the trait as protein structure, the ‘focal niche’ as the protein function, and the phylogeny as one of molecules rather than species. Perhaps digital genomic information about the number and types of different mutations in allelic variants can also be aggregated into a semi‐quantitative variable that can be evaluated using, for example, the Wheatsheaf index.

## VII. CONVERGENCE AT DIFFERENT LEVELS OF LIFE

We now consider how levels of organisation in terms of form and function might be approached in studies of convergence, and use this to propose an approach to the quantification of convergence across life (we refer readers also to Losos, 2011).

**Figure 5:**
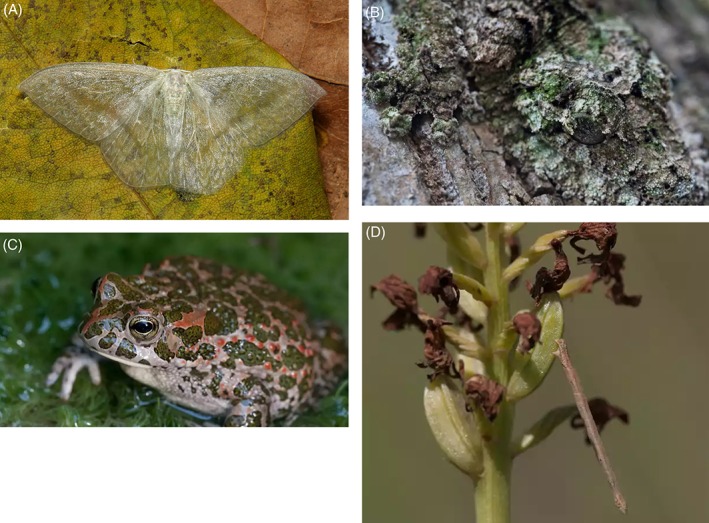
Examples of different kinds of animal camouflage. (A) Transparency in the drepanid moth *Deroca hidda* (Drepaninae). (B) Background matching by the mossy leaf‐tailed Gecko, *Uroplatus sikorae*. (C) Likely disruptive patterning on the Balearic toad, *Bufo viridis*. (D) Many caterpillars (Lepidoptera) resemble twigs, a form of camouflage known as masquerade. Here the caterpillar is somewhat out of its protective habitat, away from the twigs that it mimics. Photo credits: (A) John Hortsman/‘“itchydogimages” on Flickr’; (B–D) Michael and Richard Webster.

A number of authors have argued that convergence should be considered to operate at different levels of life (Doolittle, 1994; Losos, 2011; Maeso *et al.*, 2012; Rosenblum *et al.*, 2014), perhaps most obviously with a familiar division between form *versus* function (see a vast range of examples in McGhee, 2011). To illustrate a more detailed view on convergence in a trait at different levels of life, we take animal camouflage as an example. For the purposes of this discussion, we define camouflage in a general sense, in which animals take on some colouration (or in transparency an absence of pigmentation) which reduces their conspicuousness to predators (Fig. 5) rendering them ‘cryptic’ (Stevens & Merilaita, 2011). Camouflage is a useful example because it is straightforward to depict different levels of organisation (Table 3).

**Table 3:** A set of potential levels at which convergence can take place. ‘A’ levels represent those form/function traits that are typically considered as ‘true’ convergence (*versus* parallelism), whereas ‘B’ levels represent those developmental/mechanistic‐type traits that may be considered to be either convergent or parallel evolution. We note that, of course, with any typology such as this some levels will not apply to (or be useful for) certain systems, but individual researchers can adjust the framework to suit their own study. We set Function as the highest level, and work downward

Level	General features	Camouflage example	Type of data
(A1) Ultimate function	Ecological/evolutionary benefit provided to the organism	Reducing number of costly encounters with predators by prevention of detection	Categorical
(A2) Proximate mechanism	The general mechanism by which the function is fulfilled	One of several alternative mechanisms to achieve camouflage, e.g. transparency, background matching, disruptive colouration	Categorical
(A3) Form – physical properties	Physical/structural properties comprising a ‘form’	Spectral reflectance properties of the colour patterns	Generally quantitative
(A4) Form – chemical composition	Chemical composition of the trait which provides physical properties in A3	Molecules used as pigments; spatial arrangement of the pigment molecules in the epidermis	Categorical and quantitative
(B1) Development/maintenance	Metabolic pathways, cell specialisation, and similar mechanisms responsible for the development of a trait	Developmental sequence by which colour pigments are deposited and maintained	Categorical and quantitative
(B2) Proteome	Contains sublevels of amino acid sequence and (e.g. tertiary) structure of the protein molecules	Variation in tertiary structure of Pmel17 protein involved in melanin deposition in melanocytes	Categorical and quantitative
(B3) Genome/transcriptome	DNA sequence (and possibly epigenetic factors)	Variation in mc1r gene underlying some elements of melanin‐based colouration	Categorical and quantitative

Working across levels in the table we start with the highest level, ultimate function (A1). The general function of camouflage is to prevent detection by enemies, which contributes to fitness by raising individual survival rates. The mechanism by which the trait achieves this general function (here level A2, the proximate mechanism) is, however, variable. There is in fact a large range of mechanisms to achieve this ultimate function (Ruxton, Sherratt & Speed, 2004; Stevens & Merilaita, 2011) including transparency (Fig. 5A in which the background can be seen through the organism), background matching (Fig. 5B, in which the colour pattern of the animal matches its background preventing detection), disruptive colouration (Fig. 5C, in which colouration prevents predators detecting the boundaries of an animal's form), and masquerade (Fig. 5D, in which prey mimic non‐food items like twigs and stones).

Hence we can specify a proximate mechanism that provides the ultimate function, in our example the type of camouflage which provides the benefit of reduced detection by predators, for which there are many routes to this function. Next we have the form of the trait itself. Depending on the perspective taken and the system in question it may be appropriate to distinguish the physical and behavioural properties of the trait (A3) from its chemical composition (A4). In our example of animal camouflage, physical form can be measured as reflectance spectra of the colouration of a cryptic animal. Two cryptic patterns could in principle be similar in physical reflectance properties, but be composed of chemically quite different pigments that are distributed throughout the epidermis in different ways.

Beneath the levels discussed above are the developmental systems that underpin the phenotypes and functions in question (B1, B2, B3). These may be subject to genuine *de novo* convergence, as well as parallel evolution in homologous systems (see review in Rosenblum *et al.*, 2014). Indeed, parallelism is often defined in a way that is consistent with convergence at the genetic (B3) or protein (B2) levels as considered here. With this view we could perhaps consider parallelism as a concept that is not distinct from convergence *per se*, but simply a term used to refer to convergence at particular levels of life. In other words, terminological disputes between these two phenomena could be resolved (or at least tempered) by this realisation and more explicit reference to the level that we are referring to where relevant. See also the relevant discussion in Losos (2011).

The general function of camouflage is almost certainly widely convergent, whereas the forms that camouflage takes, both the proximate mechanisms and the phenotypes within those mechanisms, are highly divergent. This variation could conceivably be explained by phylogeny, but it is likely to also be explained as adaptive responses to variable environments. Because of different characteristics of light refraction in air and water, transparency as a means of camouflage, for example, functions better in aquatic than terrestrial habitats. Particular forms of background matching and masquerade work best when they are sited in a visual context that they resemble, and so on. Hence with camouflage there is widespread convergence in general function, but many different mechanisms and precise phenotypes are used to fulfil this general function. There is no single way to be camouflaged, hence there are locally adaptive solutions to fulfil the generally convergent function.

Finally, we propose that a useful way to think about the predictability of evolution is to evaluate the extent of convergence (both frequency and strength, using the metrics described in Sections III and IV) for traits in species that inhabit diverse environments (and more generally diverse selective contexts). A trait that is similar and convergent at all levels (in the terms of Table 3, A1–A4) across many environments is then qualitatively different, and more constrained, than one that is, for example, convergent only in general function (A1) and not at any other level.

## VIII. CONCLUSIONS

1. We need to work towards a new quantitative framework which measures the frequency and strength of phenotype convergence (such as by using the metrics reviewed here) while also taking account of variation in environmental characteristics in a quantitative manner.
2. The most convergent traits in this view, where life is most predictable, are those that evolve most often and in a most similar way across a wide diversity of selective contexts and levels of life. In addition to taking a more quantitative view, we also highlight that further work is required by method developers to continue to improve the tools available for such studies.
3. Nevertheless, recent developments have started to bring an old concept (convergence) into maturity as a rigorous and modern science.
